# A novel, semi-automatic procedure for generating slow change blindness stimuli

**DOI:** 10.1093/nc/niae004

**Published:** 2024-02-09

**Authors:** Haley G Frey, Lua Koenig, Biyu J He, Jan W Brascamp

**Affiliations:** Department of Psychology, Michigan State University, 316 Physics Road, East Lansing, MI 48824, United States; Neuroscience Institute, New York University Grossman School of Medicine, 435 East 30th Street, New York, NY 10016, United States; Neuroscience Institute, New York University Grossman School of Medicine, 435 East 30th Street, New York, NY 10016, United States; Departments of Neurology, Neuroscience & Physiology, Radiology, New York University Grossman School of Medicine, 435 East 30th Street, New York, NY 10016, United States; Department of Psychology, Michigan State University, 316 Physics Road, East Lansing, MI 48824, United States

**Keywords:** change blindness, slow change blindness, awareness, visual perception, stimulus generation

## Abstract

Change blindness is the phenomenon that occurs when an observer fails to notice what would seem to be obvious changes in the features of a visual stimulus. Researchers can induce this experimentally by including visual disruptions (such as brief blanks) that coincide with the changes in question. However, change blindness can also occur in the absence of these disruptions if a change occurs sufficiently slowly. This “slow” or “gradual” change blindness phenomenon has not been extensively researched. Two plausible practical reasons for this are that there are few slow-change stimuli available, and that it is difficult to collect trial-specific responses without affecting expectations on later trials. Here, we describe a novel, semi-automatic procedure for quickly generating many slow-change stimuli. This procedure creates stimuli that have been specifically designed to allow assessment of change blindness on individual trials without influencing subsequent trials. We include the results of three validation experiments that demonstrate that these stimuli are effective and suitable for use in systematic studies of slow change blindness.

Research in the past 30 years has studied “classic” change blindness to learn more about visual attention, visual representations of the world, and consciousness (for a review, see Simons and Levin 1997, Simons and Rensink 2005). For instance, the phenomenon has helped clarify how impaired focal attention affects the formation of memory representations, detection performance, and, indeed, conscious perception of the world around us. Normally, a sudden change in the environment produces a visual transient that automatically draws attention and allows an observer to effortlessly detect the change. Classic change blindness draws on the notion that this automatic pull of attention can be interrupted if the sudden change coincides with another visual event, such as a brief stimulus interruption or an eye movement, rendering an observer effectively blind to the change (Rensink et al. 1997, O’Regan et al. 1999, 2000, Hollingworth and Henderson 2002). Even without any such distracting visual events, observers can still be unaware of large stimulus changes if these changes occur sufficiently slowly (Simons et al. 2000, David et al. 2006). In these slow change blindness examples, the slow rate of change evades attention-drawing visual transients, and the change remains undetected.

In contrast to the relative abundance of research involving “classic” change blindness, there is a dearth of literature regarding “gradual” or “slow” change blindness (referred to as “slow change blindness” from here on). This is unfortunate because change blindness in general has proven to be such a useful paradigm for the study of visual function, and slow change blindness specifically has properties that render it suitable for investigating questions not easily addressed using classic change blindness paradigms (see Discussion). There are several practical reasons that explain the relative shortage of slow change blindness research. First, there is a lack of slow-change stimuli. Aside from a handful of classroom demos (which participants may be already familiar with), there are only a few published examples of slow-change stimuli—not enough for many experiment designs. Second, it is difficult to prepare participants for reporting changes they may notice without giving away that a slow change may occur. Third, slow change blindness can be such a robust phenomenon that experimenters run the risk of confusing participants who consistently fail to notice any changes that they are supposed to report. Fourth, and related to both previous points, it is a challenge to present successive trials to a participant, especially in designs that require the experimenter to evaluate whether changes were noticed in individual trials. This requirement is common to many appealing designs: for instance, conceivable neuroimaging designs would require data averaging across many trials that each involve blindness to the change. Not only are there currently too few stimuli to avoid repetitions, but asking about noticed changes after each trial also runs the risk of spoiling the phenomenon on subsequent trials by cueing participants to explicitly notice slow changes, as well the risk of frustrating participants who do not notice any changes. Consistent with this assessment, of the few studies that have investigated some form of slow change blindness, one study used only a single trial to avoid any order effects (Hollingworth and Henderson 2004), and another, which did include multiple different trials, found that change detection rates were increased in later trials (David et al. 2006).

To help unlock the potential of the slow change blindness phenomenon for the study of visual function, we developed a novel pipeline to quickly create many slow-change stimuli. To address the issues of assessing change blindness on individual trials and keeping the experiment engaging, these stimuli include some more obvious, quick changes in addition to the slow change of interest. This allows experimenters to ask about any noticed changes on each individual trial—a question that should prompt participants to report both quick and slow changes if they noticed them, but that does not point specifically to slow changes. We performed several validation experiments to establish the effectiveness of these stimuli for inducing and studying slow change blindness. In doing so, we found that the stimuli (i) were quick and simple to generate even in large numbers, (ii) reliably worked to evoke slow change blindness in participants, (iii) allowed us to ask about changes without revealing the nature of the slow changes to the participant (i.e. without “giving it away”), and as a result (iv) could be presented in succession with no effect of order: change blindness remained effective for later stimuli even when asking about noticed changes after each trial. Here, we present our stimulus generation method as well as the results of our validation experiments.

## Creating slow-change stimuli

To best explain this procedure, we will walk through the creation of an example stimulus. Many parameters described here (such as stimulus duration, the number of quick changes, etc.) can be easily adjusted as needed. Our current procedure generates 20-s-long videos where the main feature is a slow color change in a large, centrally located object or surface in the scene. We chose a change in color as the slow-change feature because it was a clear feature to report on, easy to manipulate in images, and avoided any suspicious partial stimulus states that might occur during a morph (in other words, every intermediate state in the color change was just as plausible a stimulus state as both the beginning and ending frames). Further, the central location of the color renders it unlikely that it goes altogether unnoticed by participants (Cohen et al. 2020). Each video also contains 1–3 quick changes, designed to be noticed more easily. This allows us to give the participants a clear task and response instructions without giving away that a particular change will occur, or even that they are missing anything at all. The resulting videos begin with 2 s of the initial stimulus state; no changes occur during this time, and the colored element remains its initial color. Over the next 16 s, the colored element undergoes a slow fade from its initial color to a final color, by adjusting the monitor’s red, green, and blue (RGB) values at a constant rate. The quick changes, each unfolding over a period of 1 s, take place at randomly generated moments during these 16 s. Each video ends with 2 s of the final stimulus state, without any further changes and with the colored element shown in its final color. We generated these stimuli using the following three steps.

### Step 1

**Figure 1.**
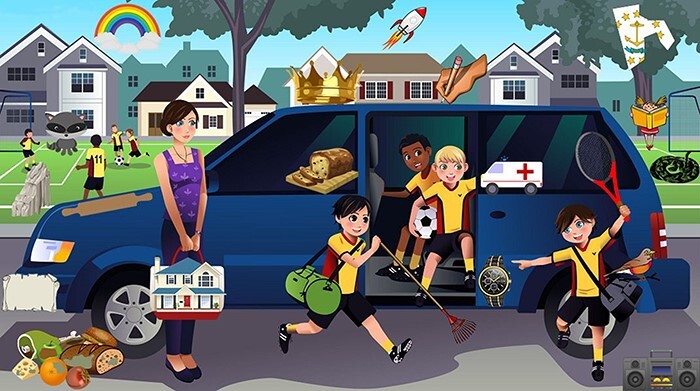
An example image that would work well as the basis for a slow-change stimulus becuase of the ease of manipulating the color of the large, central, car (while still fitting well with the scene) and other objects which can be edited for quick changes. Artisticco/Shutterstock.com

We manually collected cartoon images containing at least one large, colored area. Cartoon images work well for these stimuli because they are easy to edit (see next step) and because the large colored element will make sense in the scene even when multiple bright colors are used (see Fig. 1).

### Step 2

**Figure 2.**
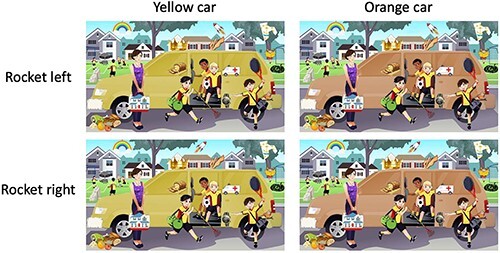
Illustration of Step 2 in our pipeline: the set of composite images that would be saved out to create a slow-change video with one quick change (rocket left to rocket right) in addition to the slow-change (yellow, versions on the left, to orange, versions on the right). Artisticco/Shutterstock.com

The large, colored area was manipulated in Adobe Photoshop (24.1.1), resulting in two versions of the image: one with the area in its initial color and one with the area in its final color. There are many workflows within Photoshop and other graphics applications which can be used to edit images for this purpose, but here we describe the workflow used in our pipeline. First, the object or area of interest was selected using the “Quick selection” tool (in Fig. 1, this was the car). Next, with this item still selected, a new adjustment layer was added by selecting “Layer -> New Adjustment Layer -> Hue/Saturation” from the menu bar at the top of the screen. With this new layer added, any adjustments will only affect the selected area. The color of the selected area was then changed using the “hue,” “saturation,” and “lightness” slider options for the layer. To create the additional, quick changes, other features of the image were also edited to create two versions (e.g. adding/removing an object, rotating/reversing an object, changing features/identity of an object). The “Quick selection,” “Brush,” and “Lasso” tools as well as the “Free Transform” option were used most often to achieve the manipulation of the quick-change features. Each of the changed feature states were isolated on their own layer of the Photoshop file so that creating composite versions of each combination of features could be done easily by toggling different layers on or off (layers in graphics applications are groups of visual elements that allow the user to organize their artwork into separate sections that can be independently manipulated). Once the desired number of changes and edits were made, each version of the image—one for each combination of features—was saved and named according to a predetermined naming pattern. For our pipeline, we chose “Img#_Color_quickChangeState.jpg” where images with additional quick changes would have additional “quickChangeState” components in the name. The corresponding code expects this naming pattern and uses the quick-change states to match pairs of images. For a video with one slow change and one quick change, four versions would be saved (two options for the slow-change feature, two options for the quick-change feature; Fig. 2); for 1 slow change and 2 quick changes, 8 versions would be saved; and for 1 slow change and 3 quick changes, 16 versions would be saved.

### Step 3

Next, we ran a script that created the slow-change videos based on those composite files. The script is available at https://github.com/haleyfrey/creating_change_blindness_stimuli.git and its full functioning can be reviewed there. The script works by (i) reading in all versions of a given image (created as described above), (ii) creating a series of frames for each quick-change state in which the colored area progressively changes from the initial to the final color (we refer to this as a “morph series” later on), and (iii) from those series, selecting a sequence of frames to be converted into a slow-change video.

**Figure 3.**
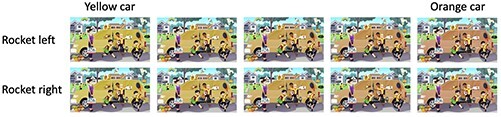
Illustration of Step 3b in our pipeline: generating the initial series of morphs between the two colors of the slowly changing element (between a yellow car, left, and an orange car, right), and the two sets differ with regard to the properties of the quickly changing element (the rocket tilted left in the top morph series, or right, in the bottom morph series) Artisticco/Shutterstock.com

**Figure 4.**
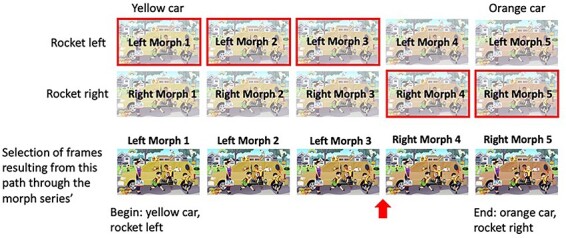
Illustration of Step 3c in our pipeline: the script uses the series of morphs illustrated in Figure 3 (the same series’ shown in the top two rows here) to choose frames that introduce a quick change (the rocket suddenly changing direction) while continuing the smooth slow change (the car color changing from yellow, left, to orange, right) will eventually form the slow-change video. Artisticco/Shutterstock.com

More specifically, for each given combination of options for the quick-change features, the script reads in the two image files that have different colors for the slow-change element (the start color and the end color) and creates a series of images that gradually morphs between the two colors by linearly interpolating the two images’ RGB values (Fig. 3). The number of images in the morph series is determined by the number of frames desired in the resulting video (this is also dependent on the desired video length and the desired frame rate of the video). In our current pipeline, we create 192 morph frames which, at a frame rate of 12 frames/s, means that the slow color change lasts 16 s in the final video. From the resulting set of parallel morph series (one for each combination of quick-change features), the code then selects a frame sequence to be used in the video. Specifically, as the script steps through the stages of the slow color morph, it adds the morph frame from the morph series containing the appropriate quick-change combination at each step. Using the example morph series’ in Fig. 3, the script may begin to step through the stages of the slow color morph from yellow to orange using the top series of images in which the rocket is tilted to the left (these “selected” frames are outlined in red in Fig. 4). When it is time for the change to occur (as determined by a random generator), the code continues to select frames that correspond to the appropriate points in the color morph, but from the series of images in which the rocket is tilted to the right (Fig. 4). The quick changes are currently designed to be non-instantaneous, lasting 1 s each in the final video. We reasoned that such quick, yet non-instantaneous changes might be easier for participants to describe than instantaneous ones, because for instantaneous changes a participant may well notice the transient but remain oblivious as to how the display looked before the transient. Once the list of selected frames is finalized (bottom row of Fig. 4), the script utilizes Ffmpeg (Tomar 2006; https://ffmpeg.org/) to convert the assembled series of images into a video. As mentioned above, we used a frame rate of 12 frames per second so that the slow color morph lasts 16 s. To avoid changes occurring too close to the beginning or end of the video as well as to ensure that participants had time to register the initial and final states of the video, the script bookends the 16 s slow color change component of the video with 2 s of the unchanging initial frame and 2 s of the unchanging final frame (before and after the slow color change, respectively), resulting in a 20-s-long change blindness video. This code can be used to generate morphs between any two colors. For example, if at Step 2, one has created versions where the slow-change element is purple, blue, orange, and yellow, then one can create morphs between purple and blue, purple and orange, purple and yellow, blue and orange, blue and yellow, and orange and yellow simply by moving the pairs of images with the desired colors into the script’s input folder.

A total of 45 stimuli generated using our pipeline, as well as the script implementing the automatized part of the pipeline, are available at https://github.com/haleyfrey/creating_change_blindness_stimuli.git.

## Validation experiments

We performed several validation experiments to verify whether the stimuli we generated were effective at inducing slow change blindness and, if so, whether they allowed participants to complete several trials in a row without the change blindness effect decreasing over repetitions. A sample of the experiment code used to present these images is available at https://gitlab.pavlovia.org/freyhale/demo_single_slow_change_stimulus_presentation

### Participants

For each of the validation experiments described below, participants were recruited using Prolific (Palan and Schitter 2018; www.prolific.co) and the experiments were made available online using Pavlovia (Peirce et al. 2019; https://pavlovia.org). Participants were paid approximately 9$/h for their participation (the rate of pay for each experiment differed slightly and was based on Prolific’s payment recommendations at the time of the experiment in question). The study was approved by the Michigan State University Institutional Review Board and was conducted in compliance with the Declaration of Helsinki. Informed consent was given by each participant through an online form via Qualtrics (www.qualtrics.com). Participants self-reported normal or corrected-to-normal vision and hearing, English fluency, and age between 18 and 65 years. Participants who had fewer than 10 previous submissions on Prolific, a Prolific approval rating below 95, or who had participated in previous iterations of our study on Prolific were not invited to participate in the study. As such, observers could only participate in one of the following studies, resulting in an independent group of observers for each experiment. Otherwise, no restrictions were imposed on participation. Participants were instructed to complete the experiment in a single sitting using a desktop or laptop computer. There was no direct control of participant environment and behavior because the study was administered online, but participants were instructed to sit at their normal viewing distance and to avoid large movements during the study. Before the study began, participants completed a blind-spot identification procedure and a debit card scaling procedure (Brascamp 2021) so that we could estimate viewing distance, as well as the size and aspect ratio of the pixels. Using this information, we adjusted stimuli so that they would appear square and subtend approximately 20° of visual angle (dva) from the participant’s viewpoint.

### Experiment 1—one slow-change stimulus

**Figure 5.**
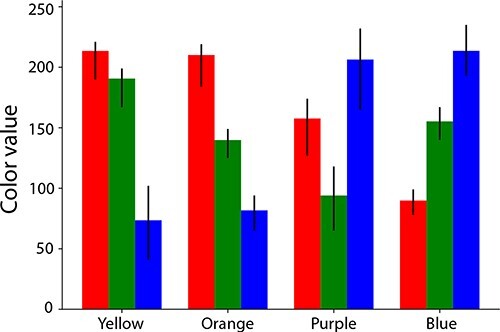
A breakdown of the average RBG values across the 11 images after attempting to standardize them; black lines span the minimum and maximum for each color value across the 11 images and the red (left), green (middle), and blue (right) bars reflect red, green, and blue color values, respectively

Our first goal in creating these stimuli was to ensure that the slow color change went unnoticed. The purpose of this experiment was to verify that participants, indeed, experienced slow change blindness while viewing one of our generated stimuli. Across four studies, we collected data from 1087 participants. Empty or incomplete data files, as well as data from people whose credit card scaling procedure was not consistent with square pixels (indicating that they did not complete the scaling procedure well and that as a result, stimuli were not presented as intended), were excluded from analysis, resulting in 923 data files for analysis. Each participant completed a single trial where they viewed a single slow-change video. Across participants, we tested 55 videos total: we used 11 different images as the basis for our videos (see “Step 1” above) and for each of those images we created 5 videos, each involving a different color combination for the initial and final color of the slow-changing image element. One combination, termed “purple–orange, non-standard” involved a purple and an orange that were unique to each of the 11 images, because they were kept consistent with the brightness and saturation of that image. As a result, the brightness and saturation values used differed substantially between the 11 images. For the other four combinations, the colors were standardized across the images. To standardize colors across images, first a target RGB value for each color was determined. Then, in Photoshop, the “Brush” tool was used to draw a patch in the desired color. Finally, the hue, saturation, and lightness of the adjustment layer (described in Step 2 of Creating Slow-Change Stimuli) were adjusted until the object color visually matched the swatch. This process was repeated for each color and for each image. Two of those combinations involved colors that are adjacent on the color wheel (yellow–orange and purple–blue); the other two involved colors that are not (purple–orange and blue–yellow). Given that these images were presented to participants using a variety of monitor types, we could not fully control for color and made no attempt to calibrate the colors. For instance, it is unlikely that different colors had the same luminance level on all, or even the majority, of the participants’ monitors. However, we did strive to match colors across images (described above). To verify our standardization, we averaged the RGB values across all pixels that made up a given image’s large, central, colored object at the start or end of a video (those pixels did not all have the same color value, because we preserved structure within the large area; see, for instance, the car in Figs 1–4). Figure 5 summarizes these average RGB values for all images, with each bar showing the across-image average, and black lines showing the across-image extremes rather than confidence intervals. Given that these lines are small, this demonstrates that each given color was roughly the same in each image. This figure also quantifies which colors we used in terms of their RGB values.

Aside from standardizing colors in this fashion, we also verified that the members of each color pair were easily discriminable when they alternated on the screen in an abrupt, rather than slow, fashion (experiment detailed below).

For each color pair, the order in which the colors appeared was fixed, and this order is indicated by the designation we use for the stimulus. For instance, for a stimulus termed “yellow–orange” the slow-change area started off yellow and gradually faded to orange.

Participants were instructed that they would see an image for 20 s and that some features of the image might change while they were looking at it. They were told to look for changes and that they could look anywhere in the image. They were not told about different types of changes (slow/quick), nor were they given examples of possible changes. As such, it is reasonable to expect the instruction to prompt participants to report both fast and slow changes, if they noticed them. After the video ended (20 s later), participants were asked whether they had noticed any changes. If yes, they were prompted to describe any changes they noticed in the provided text box. Participant responses were then manually coded to determine which changes had been detected. Reported changes were categorized as a quick change, a slow color change, and, occasionally, a change that could not be identified by the experimenter (such as a description of a change that did not occur). For the quick changes, any response that correctly identified the object that changed (e.g. the radio) or the location of the change (e.g. on the bottom right) was marked as an instance of “noticing” the quick change. For the slow color changes, any response which mentioned a change in the element that underwent the color change, even if color was not explicitly mentioned, was marked as an instance of “noticing” the slow change.

#### Analysis

Responses obtained from participants consisted of a “yes/no” response to the question of whether they noticed any changes, as well as a text box response explaining the changes noticed accompanying each “yes” response. Given that our response of interest was whether or not the participant noticed the slow color change, we needed to verify which change(s) the participant was referring to. These videos included both quick and slow changes so a “yes” response did not necessarily mean the participant noticed the slow change of interest. For this purpose, text box responses were manually coded. We employed a liberal criterion when scoring text responses: a text response that mentioned any change in the area that underwent a slow change was marked as an instance of noticing the slow change, even if the response did not mention anything about color or the change’s slow nature. Table 1 includes examples of responses and how we scored them.

**Table 1.** A set of example participant free responses and how they were coded to determine whether the slow change was detected.

Participant response	Noticed slow change
“A new window appeared”	No; while the participant correctly noticed the quick change of the window appearing, their response did not include any indication that they noticed the floor changing colors.
“the color of the background changed, a study light disappeared and reappeared, the clock on the wall disappeared and the bear’s facial expression changed to a smile.”	Yes; the participant correctly stated that the color of the background changed.
“the floor’s color was flickering”	Yes; the participant mentioned a change in the floor, which was the slowly changing object in this image.
“I think the color of the sky was changing. it may have been getting lighter or a brighter blue”	No; the sky was not the slowly changing object in this image.
“the background went from blue to purple”	Yes; the participant correctly stated that the color of the background changed.

#### Results

**Figure 6.**
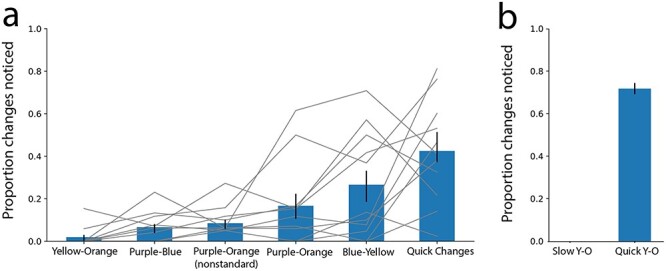
(a) Shows the proportion of changes noticed (gray lines spanning each color combination) and the average of these proportions within each color combination (blue bars) where the black bars reflect standard error of the mean across the 11 image-level proportions. The left five bars reflect the average proportion of color changes detected across the 11 images, under various color conditions and the rightmost bar reflects the average proportion of quick changes detected across the 11 images, including all color combinations. *N* = 184, 184, 188, 209, 158, 923 from left to right. (b) Compares the average proportion of yellow–orange color changes detected across three sample images when the change occurs slowly (left) or quickly (right): the left bar replots the average slow-change detection rate from the original experiment but only including the three yellow–orange stimuli used in the new control experiment (the bar is absent because that rate was 0 for those stimuli) and the right bar plots the average proportion of color changes noticed across the same three images when the color change occurs as a quick change instead of a slow change. Black bars represent standard error of the mean where samples the proportion of changes noticed for each the three images. *N* = 50, 32

Results of this experiment are shown in Fig. 6. The first five bars reflect the proportions of slow changes correctly reported for each of the color combinations used (with results averaged across the 11 images), and each gray line corresponds to one of the 11 images (Fig. 6a). The bars are ordered from left to right in ascending order based on the proportion of slow-change events that were reported. Each video contained a single slow change, so the proportion of slow-change events reported is equivalent to the proportion of participants who noticed the change. This proportion ranged from near 0 for the best color combination (yellow–orange) to about 0.25 for the worst (blue–yellow). To determine whether the combination of colors used in the morph significantly influenced detection performance, we ran a one-way repeated measures ANOVA with “color” (5 levels) as the factor. The ANOVA showed a significant effect of color combination, F(4,40) = 5.80, *P* = 0.000893. Next, we investigated whether color morphs between colors that are adjacent on the color wheel yielded smaller rates of detection than morphs between more distant color pairs. We ran a paired *t*-test between detection rates averaged across adjacent color pairs (yellow–orange and purple–blue) and the detection rates averaged across further color pairs (purple–orange and blue–yellow). Our results show that the proportion of color changes reported was lower for adjacent color combinations than for non-adjacent color combinations, t(10) = –2.68, *P* = 0.0115. Additionally, the proportion of changes detected appeared lower when we tailored the hue, saturation, and brightness of the colored area to fit with the individual images (purple–orange, non-standard), than when we attempted to match the colors across images (purple–orange), but this difference was not significant, t(10) = –1.40, *P* = 0.095. We also conducted a one-way repeated measures ANOVA with “image” (11 levels) as the factor to determine whether the detection performance was influenced by the image used. This ANOVA showed an effect of image, F(1040) = 2.72, *P* = 0.0120. In addition to the finding that some images worked better than others, the images appeared consistent in their rank of “goodness” across color combinations (see Fig. 6a for a visualization of this trend; see Supplementary Materials for more details about the individual images). However, even our worst images yielded low rates of slow-change detection when the better color combinations were used. Each color combination had at least two images where the detection proportion was 0, meaning that all observers missed the change (the average number of observers per image-color combination was about 17).

One possible explanation for the low rates of slow-change detection is a lack of engagement in the task or lack of compliance with task instructions. Another is that participants who do notice the slow change may forget about it before they have to give their response. Analysis of the participants’ quick-change detection performance provides some evidence against such factors being of major importance here. The rightmost bar reflects the proportion of quick changes reported across all images and color conditions (Fig. 6a). The overall proportion of quick changes detected was 0.44 as compared to an overall proportion of 0.12 across all slow-change conditions. This evidence supports the idea that participants were at least somewhat engaged in the change detection task and complying with the instructions, and that they could remember changes long enough to report them. Of course, the slow changes may require more effort and focus to be detected, so we cannot rule out the possibility that participants would have noticed more slow changes if they had made more of an effort.

We also considered another possible reason for the low slow-change detection rates, namely that the colors in our pairs may be too similar to each other to easily tell apart. To evaluate this possibility, we ran a brief control experiment with the same procedure and instructions as above but using only 3 of our 11 images that gave the lowest detection rates in the initial experiment, and only using the color pair that gave the lowest detection rates (yellow–orange). In this control experiment, the quick changes remained the same, but the color change also occurred quickly, instead of gradually. Under the assumption that those color changes that are the hardest to spot in a slow-change setting would also be the hardest to spot when they happen quickly, in this control experiment we used only the color combination that yielded the lowest slow-change detection rates in the initial experiment. If the colors are hard to tell apart, then this quick color change should also be detected at a low rate. The results, however, show this not to be the case: Fig. 6b shows that these color changes, although extremely hard to identify when they occur slowly, were quite noticeable when they occur quickly, with a detection proportion of nearly 0.8 (detection performance in the original experiment was 0 for these three stimuli; replotted in Fig. 6b for comparison).

### Experiment 2—multiple slow-change trials

A second objective of the present study was to develop stimuli that are suitable for systematic experiments in which multiple slow-change trials are presented to the same participant in succession. This second experiment served to verify whether trials such as those of Experiment 1 can, indeed, be presented repeatedly to the same participant without impacting change detection performance. Each participant in this experiment completed five slow-change trials, each followed by an opportunity to report any changes they noticed. (This sequence of five trials was followed by a sixth trial with a different task, which was unrelated to the purposes of the present study, and which will not be discussed here.) Each participant saw five different videos across the five trials, randomly selected from among six videos that had yielded 0% detection performance in Experiment 1. We did modify the purple–blue stimuli by using a slightly bluer purple than in Experiment 1 in an attempt to further reduce slow-change detection for this color combination. In the process of implementing this slight color change, we also flipped the color order for the stimuli involving blue and purple, so that blue became the starting color. This order change was unintentional, but we do not believe it affected our results in a relevant way. We verified that this color difference was still quite noticeable when presented as a quick change, in the same control experiment described above (Fig. 6b). Indeed, when presented as a quick change, this color change was detected by 60% of participants. The six videos used in Experiment 2 are: yellow–orange image 1, yellow–orange image 2, yellow–orange image 5, blue–purple image 3, blue–purple image 7, and blue–purple image 8.

**Figure 7.**
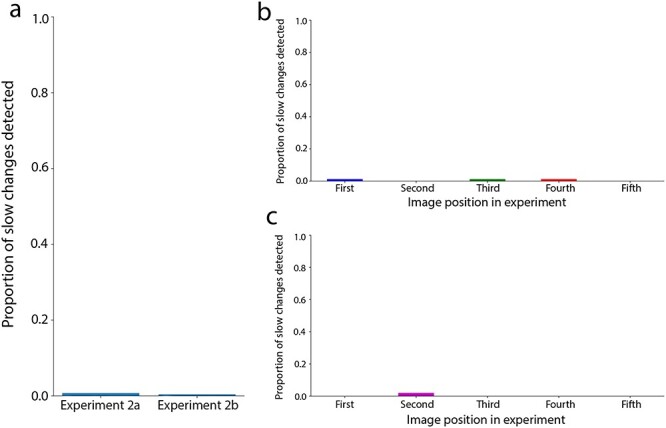
(a) Shows the proportion of slow changes detected in each iteration of Experiment 2. (b) Shows the proportion of slow changes detected at each image position in Experiment 2a. (c) Shows the proportion of slow changes detected at each image position in Experiment 2b. In (b) and (c), each color corresponds to a unique participant

**Figure 8.**
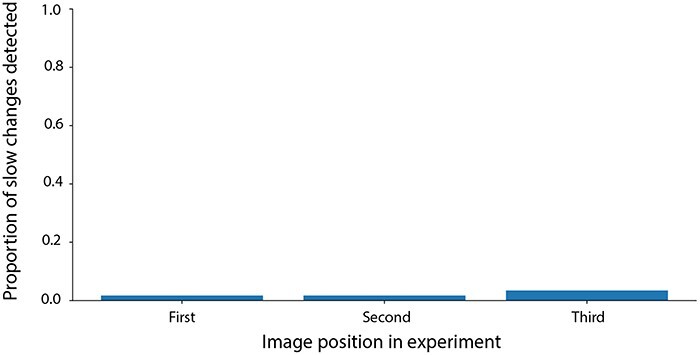
This figure shows the proportion of slow changes detected at each image position in Experiment 3: the first two bars each reflect a single unique participant, and the third bar reflects two participants who are distinct from the first two bars

We performed two versions of this experiment. For the first (Experiment 2a), 98 participants completed five slow-change trials that were set up the same as in Experiment 1. For the second (Experiment 2b), we added a fixation point to render less likely the possibility that participants simply missed the slow changes because they did not look at the right parts of the stimulus. In particular, whereas Experiment 1 and Experiment 2a had a free viewing instruction, now a white circular fixation mark was presented at the center of the image during the first 2 s and final 2 s of each video. Participants were instructed to look at the fixation mark whenever it was on the screen, but were allowed to free-view when the fixation point was removed. To help distinguish between these viewing periods, the disappearance of the fixation mark near the beginning of the video and its reappearance toward the end were accompanied by a type of “cross hair” formed by four lines (one horizontal, one vertical, and two diagonal, each a width of 5 pixels) that intersected behind the fixation mark and that each ran across the full image from one edge to the other. In addition to the visual transient to signal whether the participant could begin or end free-viewing, we reasoned it would be confusing to keep the cross-hair or fixation dot on the screen when fixation was not necessary. Given that the slowly changing colored areas of our images were all centrally located, the fixation mark increased the likelihood (limited only by a participant’s compliance with our instructions) that the participant fixated on or near the colored area, both when it had its initial color and when it had its final color. Bearing in mind that the fixation point was well within 5 dva from the colored area in 100% of our images, and that existing work shows participants’ awareness of color to be good for scene elements located close to the fovea (Cohen et al. 2020), the addition of the fixation mark served to address the possibility that participants may fail to notice a slow change because they did not look at the area of interest at the right time. We did not directly measure eye movements so we cannot assert whether participants always looked at the fixation mark when instructed, but we have no reason to believe that a large proportion of our participants ignored the fixation instruction. A total of 98 observers participated in Experiment 2a and 59 participated in 2b. Empty or incomplete data files as well as data from participants whose credit card scaling procedure was not consistent with square pixels were excluded from analysis, resulting in 82 and 50 data files for analysis, respectively.

#### Analysis

As in Experiment 1, observer responses included both a “yes/no” response as well as text responses with a description of noticed changes accompanying each “yes” response. Text responses were manually evaluated to determine whether the participant noticed the slow change of interest on each trial. Examples of responses and the evaluation are included in Table 1. Plots below include the results of the manual evaluation of the text response specifically regarding the slow color change.

#### Results

The results of Experiment 2 are shown in Fig 7. In Experiment 2a, out of 82 observers who were each shown 5 videos, only 3 individuals noticed a slow change and in Experiment 2b, out of 50 observers who were each shown 5 videos, only 1 individual noticed a single slow change. Importantly, no individual reported more than one color change. These detection rates correspond to overall detection proportions of 0.007 and 0.004, respectively (Fig. 7a). As in Experiment 1, these low detection rates were likely not due to a lack of task engagement: for quick changes, the detection proportions were 0.58 and 0.53 in the two versions of Experiment 2, respectively, so roughly 125 times greater than for the slow changes. The Experiment 2 slow-change results show that detection rates remain low, even in experiments with multiple trials. However, there is still the risk of detection rates increasing over successive trials. To address this possibility, Fig. 7b and c shows detection performance, split out across the five successive trials. Instances of change detection did not occur more frequently in later trials than earlier trials, providing no indication that participants started to “catch on” as trials progressed. Further, of the handful of participants who did report a slow change, the chances of them detecting the slow changes on subsequent trials did not increase; none of the participants noticed a second slow change, even when they first noticed a color change in the earlier trials.

### Experiment 3—only slow-change trials

The results of Experiment 1 and 2 can be explained as instances of slow change blindness where observers fail to notice the slow color change because it occurred so slowly. However, it is also possible that observers did not notice slow changes because they were not looking for slow changes. Our stimuli differed from existing stimuli that induce slow change blindness, in the presence of the quick changes. While these quick changes were included for several specific reasons (to allow a credible opportunity for the experimenters to ask for change reports; to keep participants engaged), perhaps the quick changes also had a distracting effect: they may have primed participants to look specifically for changes that happen quickly rather than slowly, and/or they may have temporarily distracted participants’ attention away from the slow change each time they happened. In other words, perhaps the quick changes led to a form of inattentional blindness rather than slow change blindness: in inattentional blindness, observers are blind to the aspects of a visual scene when their attention is directed to different aspects. To ensure that our participants’ low detection performance is not due to a distracting influence of the quick changes, we ran an additional experiment using stimuli in which one slow color change occurred and no quick changes.

In this study, 75 participants (who had not participated in our previous experiments) viewed 3 slow change images in succession. Task instructions were the same as in Experiments 1 and 2. Observers were told that while they viewed the picture, it might change, and that they needed identify whether any changes occurred, and later describe to us any noticed changes. After each trial, participants were asked whether or not they noticed any changes. If they selected yes, they were provided with a text box to describe the change in. Participants in this study performed only three trials instead of the five, as in Experiment 2, because we wanted to keep our participants engaged. We need motivated participants to obtain quality data, and we were concerned that after three videos of not noticing changes despite the task instructions, participants would lose interest and stop trying in the later trials.

#### Analysis

As in Experiments 1 and 2, observer responses included both a “yes/no” response as well as text responses with a description of noticed changes accompanying each “yes” response. Text responses were manually evaluated to determine whether the participant noticed the slow change of interest on each trial. Examples of responses and the evaluation are included in Table 1. Plots below include the results of the manual evaluation of the text response specifically regarding the slow color change.

#### Results

Figure 8 shows the results of Experiment 3. Of the 58 participants, only 4 individuals noticed the slow change. Importantly, no participant noticed more than one slow change despite being presented with multiple videos. Overall, the proportion detection of this experiment was 0.023 (4 detected slow changes out of 174). While numerically larger than detection rates in Experiment 1 and Experiment 2, a two-sample z test reveals that the rate of detection is not statistically different from the detection rate in Experiment 1 (z = –1.64, *P* = 0.101) or the detection rate in Experiment 2 (z = –1.78, *P* = 0.075), thus providing no evidence that the presence of quick changes affected slow change detection rates in our study. The rate of 0.023 also indicates that the vast majority of slow changes (97.7%) went undetected in this experiment, indicating these stimuli induce strong change blindness irrespective of the presence of quick changes. There is still a possibility that participants spontaneously adopted a quick-change strategy despite the lack of any quick changes or instructions motivating them to do so, and the consequences of this are considered in the Discussion.

## Discussion

Change blindness is a fascinating illustration of the fact that looking is not the same as seeing, and classic change blindness forms a valuable paradigm in the fields of attention and perception research. For example, it allows researchers to differentiate between what information is available to the eyes and which information the participant can report on. Slow change blindness, although as compelling as classic change blindness, does not currently play that role—probably in part due to practical difficulties, which this study set out to address. For this study, we designed a method for quickly creating slow-change stimuli and developed an experimental paradigm (which combines the slow changes with more noticeable, fast, changes) to allow systematic investigation of slow change blindness. In a set of experiments, we established that the generated stimuli successfully elicited slow change blindness and that the experimental paradigm allowed slow change blindness to be assessed on an individual trial basis without alerting participants to the presence of slow changes or confusing them with an extremely difficult task.

One unexpected pattern in our data was that some images consistently produced better slow change blindness than other images, across a range of different color pairs. Though the images each had in common a large, central item that underwent a slow color change, the characteristics of this area varied. In some images, the change area was an item such as a car, a boat, or a house that contributed greatly to the meaning of the scene. In others, the area (still large and centrally located) was the wall or floor. Perhaps these latter components of the scene are processed and perceived differently by observers, given that they make up the image background rather than directly contribute to the gist of the scene. David et al. (2006) have speculated whether the meaning of changes influences change blindness. In their study, change blindness was present (detection rate of 0.15) when highly relevant emotional faces underwent a change, but change blindness was stronger when the change was in a less semantically relevant, non-face area of the image. Not only might the semantic relevance of a scene region affect its processing in terms of attention allocation, but it may also affect the frequency and duration of fixations on the region. Studies have demonstrated that, at least for quick changes, observers are more likely to experience blindness to items that they do not fixate on (Henderson and Hollingworth 1999). Future investigations into why particular images work better over others will likely lead to revelations about why and how slow change blindness arises. Although our current dataset (obtained using videos based on 11 distinct images in total) does not allow systematic investigation of what scene characteristics make a slow-change stimulus effective, the experimental tools we provide make such future investigations possible.

While our data do not reveal much about the role of scene properties, they do reveal how the choice of the slow change colors impacts change blindness (Fig. 6a). Our main observation is that a transition between two colors leads to fewer detections if the colors are relatively close together on the color wheel. There are a few potential reasons for this. For more distant color pairs, the rate of color change per unit time was higher in our experiments, so perhaps the change was not sufficiently “slow” to evade detection. A different but related explanation is that to morph between pairs of more distant colors in our design, the resulting intermediate RGB values resembled another nameable color. For example, the transition from purple to orange appeared distinctly “pink” for a few moments, and this shade was often what participants mentioned if they reported this color change. In contrast, the transition from, say, blue to purple did not involve such intermediate shades with different naming. It is possible that this presence or absence of an additional color naming boundary during the morph contributed to a color change being noticed more frequently. (Note that, given that the intermediate colors were determined by averaging RGB values, the transitions pass through the shortest RGB path—which is not necessarily the same as the shortest path along the color wheel.) Finally, we were unable to control for brightness during this experiment because observers participated on their own monitor under a variety of conditions. It is possible that luminance changes were larger for more distant pairs of colors, and that this contributed to the higher rates of slow change detection in certain color pairs.

The effectiveness of change blindness in the present study compares favorably to that reported in previous work. We know of two other studies that reported such data for slow color changes (Simons et al. 2000, David et al. 2006). The proportion of reported slow changes that we observed is similar to what was reported in the study by David et al. (2006), and even somewhat lower than what was found by Simons et al. (2000). Of note, compared to our stimuli, in the David et al.’s (2006) study, the scene elements undergoing a color change were generally smaller, less centrally located, and less relevant to the meaning of the scene (a person’s clothing item or a background item), so in that sense, the similarity in detection performance across both studies is noteworthy.

One notable difference between our stimuli and those in existing studies is the presence of quick changes in addition to the slow changes themselves. While this was a deliberate design choice (see above), it is conceivable that the quick changes also had the unintended side effect of rendering the slow changes less reportable. For instance, while a participant focuses their attention on the location of a recent quick change, the ongoing slow change may be more likely to escape attention than it already was, effectively inducing a type of inattentional blindness. If the quick changes played a substantial role in this regard, we would expect the proportion of reported slow changes to be lower for videos that contained more quick changes (this number varied from 1 to 3 in our design). However, when correlating the 11 videos’ detection rates with their numbers of quick changes, we found no evidence for such an influence (*r*^2^ = 0.027, *P* = 0.63), suggesting that the presence of quick changes likely does not transiently affect participants’ ability to detect slow changes around the time of the quick change. The results of Experiment 3 also provide support for this conclusion. In addition to potentially attracting attention away from the slow change at the moment that the quick change occurs, the presence of quick changes may also induce a longer-lasting tendency for observers to specifically search for the easier-to-detect quick changes. In this case, inattentional blindness could arise from the attentional strategy used for detecting quick versus slow changes, rather than any distracting aspect of the quick changes as they occur. The results of Experiment 3 show that such an influence, if it is present, is not the explanation for the low slow change detection rates in our study: participants’ rates of detecting slow changes are similarly low, regardless of whether any quick changes are present.

On a more conceptual note, slow change blindness as a phenomenon is defined operationally as a failure to report changes in a scene that is attentively observed, if those changes occur slowly. Determining the mechanism behind this phenomenon is an open question and it is entirely possible that the processes involved overlap with those involved in forms of inattentional blindness. For example, people may naturally employ a search strategy that is suboptimal for detecting slow changes. Such considerations relate to the slow change blindness phenomenon in general rather than to our approach to studying it. Regardless, our approach may help investigate slow change blindness, inattentional blindness, and their relationship.

Slow change blindness is an appealing topic of study not only because it is less well understood, but also because of the parallels to human experience. While classic change blindness is a robust phenomenon, it is rare that such changes will be encountered in normal life. On the other hand, slow changes are more lifelike. For example, a cloud slowly passing over the sun may go unnoticed until the sun is suddenly shining again. This begs the question of how much of the visual world is represented from moment to moment. Though we “feel” as though we exist in a rich and stable world, the brain is constantly bombarded with information that we never consciously experience and may not retain as much as we would expect from one fixation to another (Blackmore et al. 1995). The present stimuli offer an opportunity to learn more about how viewers interact with the visual world, and how we reliably miss large changes that happen slowly right before our eyes.

In sum, this study presents experimental tools that enable researchers to investigate slow change blindness in systematic experiments that involve many trial repetitions or use slow-change stimuli with a wide range of visual content. As such, these results stand to further increase the value of slow change paradigms in the hands of experimentalists, and to thereby contribute to a better understanding of the slow change blindness phenomenon itself, as well as to that of visual processing, attention, and conscious perception more generally.

## Data Availability

Code to generate the stimuli and a set of example stimuli are available at https://github.com/haleyfrey/creating_change_blindness_stimuli.git. Data set available upon request. Code to demo presentation of stimuli is available at https://gitlab.pavlovia.org/freyhale/demo_single_slow_change_stimulus_presentation. Analysis code available upon request.
